# Mild encephalitis/encephalopathy with reversible corpus callosum lesion associated with listeria infection with meningoencephalitis: a case report

**DOI:** 10.3389/fmed.2025.1568219

**Published:** 2025-07-11

**Authors:** Jiahua Liang, Gang Peng, Yiyi Luo, Jiayu Tang

**Affiliations:** ^1^The School of Clinical Medicine, Hunan University of Chinese Medicine, Changsha, Hunan, China; ^2^Department of Neurology, Brain Hospital of Hunan Province, Changsha, Hunan, China

**Keywords:** *Listeria monocytogenes*, meningoencephalitis, MERS, adult patients with normal immunity, case report

## Abstract

**Background:**

Currently, *Listeria monocytogenes* (LM) meningoencephalitis is rare in immunocompetent individuals, and its association with mild encephalitis/encephalopathy with a reversible splenial lesion (MERS) is even more uncommon.

**Case presentation:**

We report a case of an immunocompetent adult female presenting with headache, fever, and vomiting, accompanied by hyponatremia and atypical splenial lesions of the corpus callosum on brain MRI. Both cerebrospinal fluid (CSF) metagenomic next-generation sequencing (mNGS) and culture confirmed LM infection, leading to a diagnosis of LM meningoencephalitis. The patient recovered completely after treatment with meropenem.

**Conclusion:**

This case highlights distinct clinical manifestations, laboratory findings, imaging features, and therapeutic responses in immunocompetent patients compared to immunocompromised populations. The atypical radiological presentation and favorable outcome underscore the importance of early pathogen identification via advanced diagnostics like mNGS. Our findings provide valuable insights into the heterogeneity of LM infections and emphasize the need for heightened clinical vigilance even in immunologically normal hosts.

## Introduction

Invasive listeriosis primarily affects individuals with compromised immune systems, pregnant women, and the elderly, leading to conditions such as meningitis, sepsis, and spontaneous abortion (1). *Listeria monocytogenes* (LM) meningitis is essentially bacterial meningitis. Clinical manifestations typically include fever, headache, focal neurological deficits, seizures, altered mental status, and elevated intracranial pressure (2). A distinguishing feature of LM central nervous system (CNS) infections is the propensity to cause meningitis, meningoencephalitis, or rhombencephalitis. In severe cases, acute hydrocephalus may develop within a few days of onset (3). LM rhombencephalitis (i.e., brainstem encephalitis) is characterized by progressive brainstem dysfunction, typically presenting in a biphasic pattern with non-specific prodromal symptoms, often leading to misdiagnosis as a clinical isolated syndrome (4). Additionally, in a small proportion of LM CNS infections, brain abscesses may develop without meningitis involvement (2). Meningitis and meningoencephalitis account for the majority (70%–97%) of human LM CNS infections (5).

Mild encephalitis/encephalopathy with reversible splenic involvement (MERS) is a distinct clinical-radiological syndrome characterized by the involvement of the splenium of the corpus callosum on brain MRI, with clinical manifestations of mild encephalitis or encephalopathy and a good prognosis (6). Meningoencephalitis caused by LM leading to MERS is extremely rare and has not been thoroughly described or studied. A systematic review including 51 adult patients with MERS revealed that 88% of patients had prodromal symptoms before neurological manifestations, mainly fever (78%), headache (50%), followed by seizures (22%) and consciousness disturbance (22%) (7). The etiology of MERS is diverse, including infections and metabolic abnormalities, with pathogens such as viruses, mycoplasma, and LM being implicated (8–10). On head MRI, MERS typically presents with high signal intensity on DWI of the corpus callosum, mild high signal on T2-weighted imaging, and isointensity to mildly low signal on T1-weighted imaging. During the acute phase, reduced diffusion is seen, and abnormal signals usually decrease or disappear after 1–3 weeks (11). The pathogenesis of MERS remains unclear, though several reports suggest an association with (131 hyponatremia.0 ± 4.1 mmol/L), which can alter brain osmolality and lead to cerebral edema, possibly representing a pathophysiological mechanism of MERS (12). Furthermore, multiple factors may contribute to the development of MERS. For instance, IL-6 levels have been found to increase in MERS patients associated with Mycoplasma pneumonia, suggesting that IL-6 might play a role in the occurrence of MERS (10, 13).

Currently, both domestic and international guidelines recommend ampicillin or penicillin as first-line treatments for LM infections (14). The European Society of Clinical Microbiology and Infectious Diseases (ESCMID) acute bacterial meningitis management guidelines suggest meropenem, trimethoprim-sulfamethoxazole, or linezolid as alternative treatments for LM infection (15). Literature evidence indicates that insufficient early treatment may be associated with higher mortality rates and a tendency for residual neurological symptoms (14). LM meningitis is extremely rare in otherwise healthy individuals. In this report, we present the case of a 65 years-old female patient with normal immune function who developed MERS due to LM infection, aiming to contribute to better research, diagnosis, and treatment of this condition.

## Case history

**FIGURE 1:**
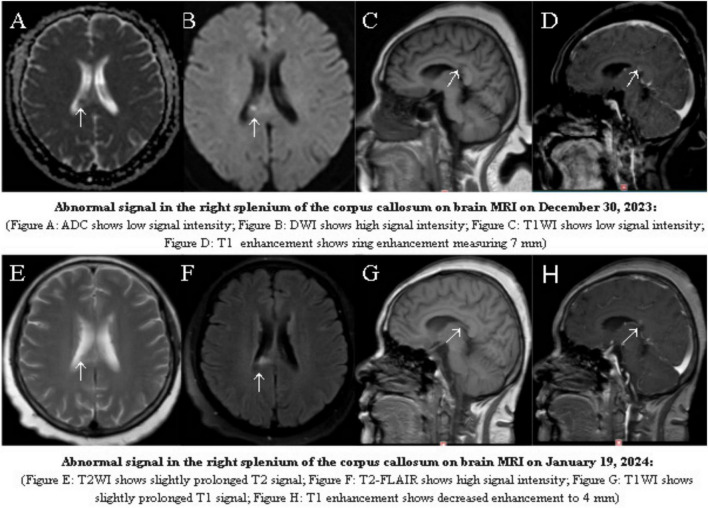
Abnormal signal in the right splenium of the corpus callosum on brain MRI on 30 December 2023: **(A)** ADC shows low signal intensity**; (B)** diffusion-weighted imaging (DWI) shows high signal intensity; **(C)** TIWI shows low signal intensity; **(D)** T1 enhancement shows ring enhancement measuring 7 mm). Abnormal signal in the right splenium of the corpus callosum on brain MRI on 19 January 2024: **(E)** T2WI shows slightly prolonged T2 signal; **(F)** T2-FLAIR shows high signal intensity; **(G)** TIWI shows slightly prolonged T1 signal; **(H)** Tl enhancement shows decreased enhancement to 4 mm).

**FIGURE 2:**
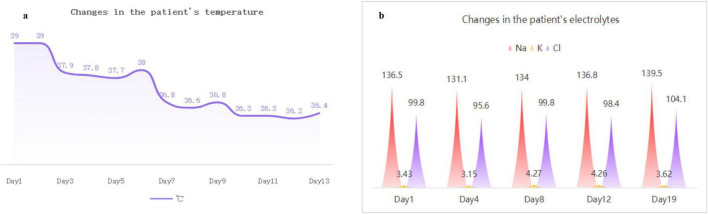
Longitudinal changes in core temperature and serum electrolytes during hospitalization. **(a)** The patient exhibited febrile fluctuations (38–39°C) during the initial week, with subsequent resolution to normothermia. (**b**) Recurrent hyponatremia and hypokalemia were documented in Week 1, resolving to standard reference ranges thereafter.

**FIGURE 3:**
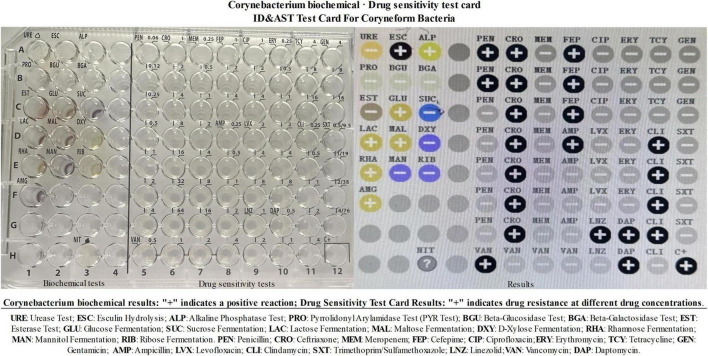
Corynebacterium biochemical results: “+” indicates a positive reaction; drug sensitivity test card results: “+” indicates drug resistance at different drug concentrations. URE, urease test; ESC, esculin hydrolysis; ALP, alkaline phosphatase test; PRO, pyrrolidonyl ary lamidase test (PYR Test); BGU, beta-glucosidase test; BGA, beta-galactosidase test; EST, esterase test; GLU, glucose fermentation; SUC, sucrose fermentation; LAC, lactose fermentation; MAL, maltose fermentation; DXY, d-xylose fermentation; RHA, rhamnose fermentation; MAN, mannitol fermentation; RIB, ribose fermentation. PEN, penicillin; CRO, ceftriaxone; MEM, meropenem; FEP, cefepime; CIP, ciprofloxacin; ERY, erythromycin; TCY, tetracycline; GEN, gentamicin; AMP, ampicillin; LVX, levofloxacin; CLI, clindamycin; SXT, trimethoprim/sulfamethoxazole; LNZ, linezolid; VAN, vancomycin; DAP, daptomycin.

A 65 years-old female patient was admitted with complaints of “headache for 5 days and fever for 3 days.” Five days prior to admission, the patient suddenly developed persistent dull pain in the forehead and occipital region, accompanied by nausea and vomiting. Two days later, she developed a fever, with a highest recorded temperature unknown, but no cough, sputum production, diarrhea, abdominal pain, abnormal behavior, or limb motor dysfunction. She was admitted to our hospital on 30 December 2023. The patient had a long-standing history of stage 3 hypertension (high-risk category) with no clear history of exposure to contaminated food or other significant medical history. Physical Examination: Temperature: 39.0°C, Pulse: 99 beats/min, Respiratory rate: 20 breaths/min, Blood pressure: 160/92 mmHg. Cardiovascular, pulmonary, and abdominal examinations revealed no significant abnormalities. Neurological examination showed that the patient was conscious, with normal higher cortical function. No abnormalities were found during cranial nerve examination. Neck rigidity was positive, and the rest of the neurological examination was negative. Laboratory Tests upon Admission: White blood cell count (WBC): 9.62 × 10^9^/L, Neutrophil percentage (NEUT%): 79.3%, Eosinophil count (EOS): 0.00 × 10^9^/L, Inflammatory markers: CRP 27.20 mg/L, PCT 0.056 ng/mL, IL-6 16.950 pg/mL, Serum electrolytes: K^+^ 2.96 mmol/L, Na^+^ 135.4 mmol/L, Routine urine test (UA), stool routine test (SE), liver and renal function tests (LFT, RFT), coagulation function (CF), myocardial enzymes (CE), BNP, and pre-transfusion tests (PTI) showed no significant abnormalities. Imaging: Brain MRI with non-contrast, contrast-enhanced, and diffusion-weighted imaging (DWI) sequences demonstrated abnormal signals in the corpus callosum, with possible enhancement of the right frontal Pia mater (see Figure 1). Based on the patient’s history and examination, a preliminary diagnosis of “intracranial infection” was considered. As the nature of the infection was unclear, potential viral, bacterial, and parasitic pathogens were to be investigated. Empiric treatment with “Ceftriaxone 2 g Qd” for infection and “Acyclovir 500 mg Q8h” for antiviral therapy was initiated, along with symptomatic and supportive treatment. Lumbar Puncture Examination: CSF pressure: 135 mmH_2_O. CSF cell count (CST): 712 × 10^6^/L, CSF white blood cell count (WBC): 657 × 10^6^/L. CSF Biochemistry: Total protein (TP): 1699.0 mg/L, Glucose (GLU): 2.39 mmol/L, Chloride (Cl): 115.8 mmol/L. Tuberculosis smear: Acid-fast bacilli negative (−), Bacterial smear: Negative (−), CSF India ink staining: Negative (−). mNGS of CSF: Result: *Listeria monocytogenes* infection (see Tables 2, 3). CSF Bacterial Culture and Identification: *Listeria monocytogenes*: Positive (see Figure 3). Antimicrobial Susceptibility Test: Sensitive to Ampicillin, Penicillin, Meropenem, and Linezolid. CSF Fungal Culture and Identification: Negative. Treatment Adjustments: Acyclovir and Ceftriaxone were discontinued, and treatment was switched to “Meropenem 2 g Q8h” for infection management. During hospitalization, the patient experienced significant electrolyte imbalance, for which symptomatic treatment to maintain electrolyte balance was administered, with repeated checkups, as shown in Figure 2. After 16 days of antimicrobial treatment, the patient had no fever or headache, with a blood test showing near-normal results. Follow-up lumbar puncture revealed: CSF pressure: 155 mmH_2_O. CSF cell count (CST): 74 × 10^6^/L, CSF white blood cell count (WBC): 74 × 10^6^/L. CSF Biochemistry: Total protein (TP): 740.6 mg/L, Glucose (GLU): 3.26 mmol/L, Chloride (Cl): 127.2 mmol/L. Tuberculosis smear: Negative (−). Bacterial smear: Negative (−). India ink staining: Negative (−). CSF bacterial and fungal cultures: Negative (see Table 1). On 19 January 2024, a follow-up head MRI with enhancement showed that the abnormal signal in the corpus callosum had reduced in size, and no enhancement was observed in the right frontal dura mater (see Figure 1). After 22 days of treatment, the patient experienced no further headache or fever, and her general condition stabilized. She was discharged upon meeting discharge criteria. Follow-up: The patient was contacted by telephone 1 year after discharge. She reported that she was living and working normally, without recurrence or any other symptoms.

**TABLE 1:** Results of cerebrospinal fluid examination during hospitalization.

Para-clinical investigation	Day 2	Day16	Reference interval
CSF appearance	Turbid	Clear	Clear
CSF pressure	135 mmH_2_O	155 mmH_2_O	80–180 mmH_2_O
CSF Pandy	(+)	(+)	Negative
CSF Cell Count	712 × 10^6^/L	74 × 10^6^/L	0∼8
CSF WBC count	657 × 10^6^/L	74 × 10^6^/L	0∼8
CSF PMNCR	11%	0%	–
CSF MNCR	89%	100%	–
CSF AFS	(−)	(−)	–
CSF IIS	(−)	(−)	–
CSF bacterial smear	(−)	(−)	–
CSF glucose	2.39 mmol/L	3.26 mmol/L	2.50–4.50 mmol/L
CSF proteins	1699.0 mg/L	740.6 mg/L	150–450 mg/L
CSF lactate	4.45 mmol/L	2.76 mmol/L	1.33∼1.78 mmol/L
CSF Cl	115.8 mmol/L	127.2 mmol/L	118–132 mmol/L
CSF microscopy	Rare gram-positive coccobacilli	Rare gram-positive coccobacilli	Absence of microbial flora
CSF culture	Listeria monocytogenes (+)	Listeria monocytogenes (−)	Negative

CSF, cerebrospinal fluid; CSF WBC count, cerebrospinal fluid white blood cell count; CSF PMNCR, cerebrospinal fluid polymorphonuclear cell ratio; CSF MNCR, cerebrospinal fluid mononuclear cell ratio; CSF AFS, Cerebrospinal fluid acid-fast staining; CSF IIS, cerebrospinal fluid India ink staining.

## Discussion

*Listeria monocytogenes* is a Gram-positive, facultative intracellular bacterial pathogen that primarily spreads through foodborne transmission. Once ingested, it crosses the intestinal barrier, leading to systemic dissemination, and has the ability to actively cross the blood-brain and placental barriers, infecting the central nervous system and the fetus (16). There have also been reports suggesting that LM can enter the human body through sporadic routes, such as the eyes, damaged skin, or mucous membranes (17). Although this patient did not report a clear history of consuming contaminated food prior to the onset of illness, she exhibited significant gastrointestinal symptoms during her hospitalization, including frequent vomiting. This suggests a high likelihood of gastrointestinal infection, though other sporadic routes of infection cannot be ruled out.

A retrospective single-center study in Poland in 2019 showed that invasive listeriosis is associated with older age, cancer, and the use of immunosuppressive agents (18). In healthy individuals, LM infections are usually asymptomatic or present as mild, self-limiting diseases (3). In immunocompromised patients, invasive listeriosis meningitis often progresses rapidly and the septicemia is accompanied by systemic symptoms such as fever and muscle aches, etc., respiratory symptoms such as cough (rarely productive), respiratory distress, etc., gastrointestinal symptoms such as vomiting and diarrhea, etc., neurological symptoms such as headache, dizziness, seizures and altered mental status, etc., (19). Although antibiotic treatment can be effective, invasive listeriosis still carry a high mortality rate. A retrospective study in Spain, analyzing 8,152 LM cases from 2,000 to 2,021, found that the mortality rate for elderly and immunocompromised LM patients increased from 39.5% in 2,000 to 60% in 2,021 (19). In the case reported here, the patient only presented with fever, headache, and vomiting, without a past medical history of headaches, fever, etc., associated with immune system disorders. These factors may have contributed to her favorable prognosis, as she responded well to targeted antimicrobial therapy and did not experience prolonged progression or complications.

In this case, imaging revealed that, in addition to meningeal involvement, the infection also affected the splenium of the corpus callosum, showing ring-enhancing signals on contrast-enhanced MRI, a rare site of infection. This suggests the possibility of mild encephalitis/encephalopathy with reversible changes in the splenium of the corpus callosum (MERS). In this case, the patient presented with fever and headache, infection-related markers such as elevated white blood cell count, neutrophil percentage, and IL-6 levels, along with persistent hyponatremia. Imaging showed ring enhancement in the splenium of the corpus callosum, which is consistent with the findings of bacterial meningitis caused by LM and MERS in a two-and-a-half-years-old infant reported by Xu et al. (10). Furthermore, after treatment, repeat MRI showed a reduction in ring enhancement and shrinkage of the lesion, consistent with the characteristics of MERS.

Bacterial culture from CSF is the gold standard for diagnosing invasive listeriosis, but it has limitations, including a low positivity rate, potential for false positives, and long culture times (13). Current evidence demonstrates that CSF cultures require ≥ 24 h of incubation to yield bacterial growth for subsequent identification, resulting in prolonged diagnostic delays. For clinical diagnosis, real-time PCR analysis of CSF demonstrates superior efficiency by enabling bacterial identification within < 4 h (20). mNGS is also an important diagnostic tool. Some studies have reported that it takes an average of 47 h from admission to obtain a positive mNGS report (21). In our case, the time from patient admission to obtaining a positive mNGS report was 51 h. Therefore, for patients with severe intracranial infections of unknown etiology, either cerebrospinal fluid mNGS or real-time PCR can aid in pathogen diagnosis and timely targeted treatment, improving patient cure rates. Following identification of LM infection in the patient via mNGS (Tables 2, 3), the bacterial characteristics were analyzed in combination with results from CSF culture, biochemical tests, and antibiotic sensitivity assays (Figure 3). The biochemical profile, including RHA(+), DXY(−), GLU(+), MAL(+), MAN(−), ESL(+), LAC(+), AMG(+), and ALP(+), aligns with the features of LM previously analyzed by Osek J. et al. (22). Additionally, the analysis revealed that LM exhibited resistance to multiple antibiotics (Figure 3). In this case, empirical antimicrobial therapy was ineffective, with severe headache and fever, persistent elevated inflammatory markers, and mNGS results indicating LM infection. Given the critical condition and the difficulty in identifying the infection, we promptly switched to broader-spectrum meropenem therapy. Following this change, the patient’s clinical symptoms, infection markers, and imaging results improved. International studies have demonstrated that the mortality rate for LM patients treated with meropenem is higher compared to those treated with benzylpenicillin or ampicillin (14). However, there are also reports recommending the use of meropenem and ampicillin for empirical treatment (5). Additionally, in a guinea pig model of LM meningitis, meropenem treatment was found to be as effective as ampicillin combined with gentamicin (23).

**TABLE 2:** The metagenomic next-generation sequencing (mNGS) identification results of cerebrospinal fluid.

Type	Name	Sequence number
**Genus**		
G+	Listeria	8,179

Source of test results: ChengduSnyan Diagnostics Co., Ltd.

**TABLE 3:** The metagenomic next-generation sequencing (mNGS) identification results of cerebrospinal fluid.

Name	Specific sequence number	Relative abundance	Coverage
**Species**			
*Listeria monocytogenes*	8,013	79.10%	22.72%

Source of test results: ChengduSnyan Diagnostics Co., Ltd.

## Conclusion

In summary, LM meningoencephalitis is exceedingly rare. Clinicians should consider LM infection in immunocompetent patients presenting with non-specific symptoms such as headache and fever, where early real-time PCR or mNGS for pathogen identification is crucial. Antimicrobial susceptibility testing tailored to LM can further optimize antibiotic selection and improve prognosis. For patients with meningoencephalitis accompanied by elevated inflammatory markers, hyponatremia, and DWI hyperintense lesions in the corpus callosum, suspicion for MERS should be heightened. A limitation of this study is the lack of strain-specific characterization analysis of the LM isolate; future research is warranted to clarify the association between LM strains and MERS pathogenesis.

## Data Availability

The original contributions presented in this study are included in this article/supplementary material, further inquiries can be directed to the corresponding author.
